# First person – Chang Sun

**DOI:** 10.1242/dmm.049796

**Published:** 2022-08-26

## Abstract

First Person is a series of interviews with the first authors of a selection of papers published in Disease Models & Mechanisms, helping early-career researchers promote themselves alongside their papers. Chang Sun is first author on ‘ Context matters – Daxx and Atrx are not robust tumor suppressors in the murine endocrine pancreas’, published in DMM. Chang is a graduate research assistant in the lab of Guillermina (Gigi) Lozano at The University of Texas MD Anderson Cancer Center, Houston, TX, USA, investigating epigenetics, transcriptional regulation, and their roles in both embryonic development and tumourigenesis.


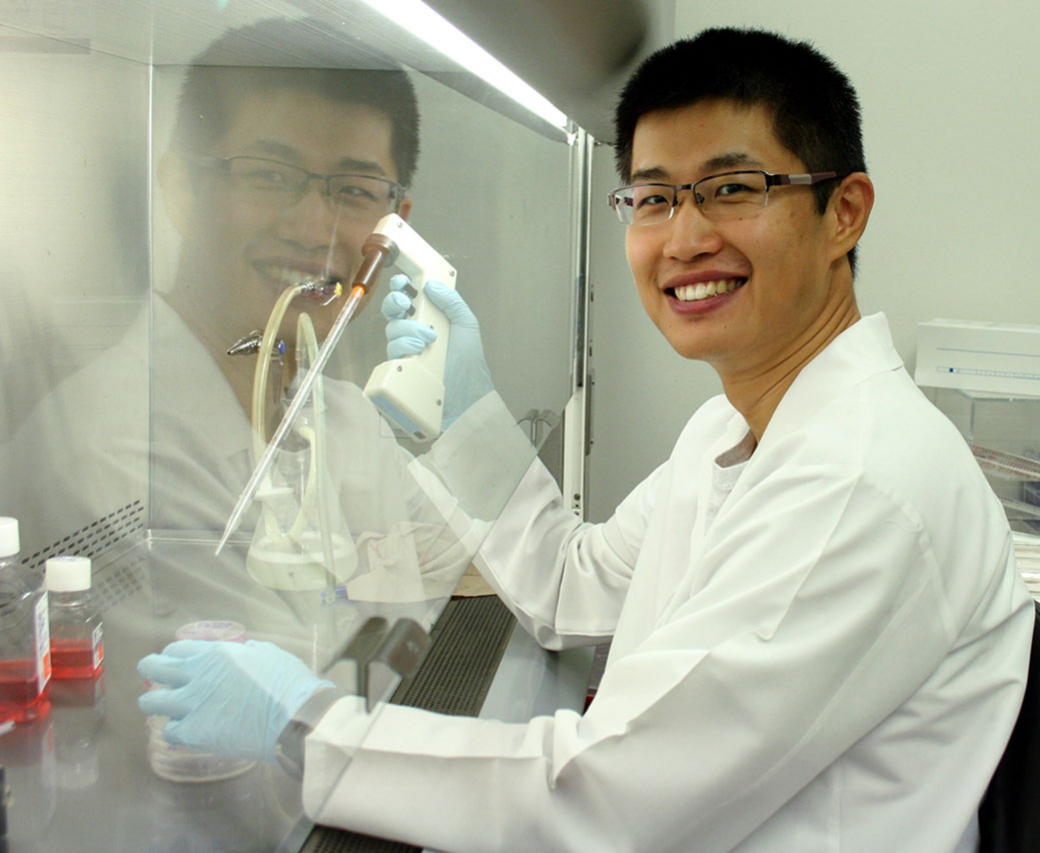


**Chang Sun**

**How would you explain the main findings of your paper to non-scientific family and friends?**

To better understand the disease development process and find therapeutical opportunities, we need model systems to recapitulate the diseases. A lack of models can hinder the research in certain diseases. Pancreatic neuroendocrine tumours (PanNETs) are one type of pancreatic cancer with a limited number of models. My work focused on building a new mouse model for this relatively rare but lethal disease. We found that it is difficult to do so potentially due to the differences between human and mouse. This suggests that some human-specific features in the genome contribute to the formation of PanNETs. Moreover, this re-emphasizes the need for developing models for the diseases.

“Daxx and Atrx are not robust tumour suppressors in the endocrine pancreas of mice. This indicates that the context of a human genome is crucial for tumourigenesis.”

**What are the potential implications of these results for your field of research?**

Mutually exclusive loss-of-function mutation in DAXX and ATRX occurs in 43% of PanNETs. This work aimed to understand tumour suppressor mechanisms through the development of genetically engineered mouse models of DAXX/ATRX mutant PanNETs. Our findings suggest that Daxx and Atrx are not robust tumour suppressors in the endocrine pancreas of mice. This indicates that the context of a human genome is crucial for tumourigenesis, thereby providing insights into the essential disease mechanisms. Specifically, the impact of human-specific features (telomere length, transposable elements) on tumourigenesis are not easily recapitulated in mice. To understand the essential disease mechanism(s) downstream of mutations in DAXX and/or ATRX, new human models are required, and could include patient-derived organoids or xenografts. It may also be instructive to evaluate Daxx and/or Atrx loss in the context of genetically engineered mouse models with telomeres that more closely resemble human, for example in the context of telomerase deficiency or in outbred strains.

**What are the main advantages and drawbacks of the model system you have used as it relates to the disease you are investigating?**

Genetically engineered mice have been used as a model system for different diseases for decades. However, there are two main drawbacks of using mice to model PanNETs with *DAXX* or *ATRX* mutations. First, inbred mice have much longer telomeres compared to humans. There is a perfect association between PanNETs with DAXX/ATRX mutations and tumour that activates the alternative lengthening of telomeres mechanism, suggesting that telomere dysfunction contributes to tumourigenesis, which may not be recapitulated as a result of the long telomeres present in inbred mouse strains. Second, the non-coding regions of the genome, including those that contain repeat elements, is not conserved between human and mouse. Daxx and Atrx play a role in silencing endogenous retroviral elements in the genome, and while de-repression is likely to be conserved between human and mouse genomes, the downstream consequences on transcriptional and cellular programmes are likely to be different and highly dependent on context.

**Figure:**
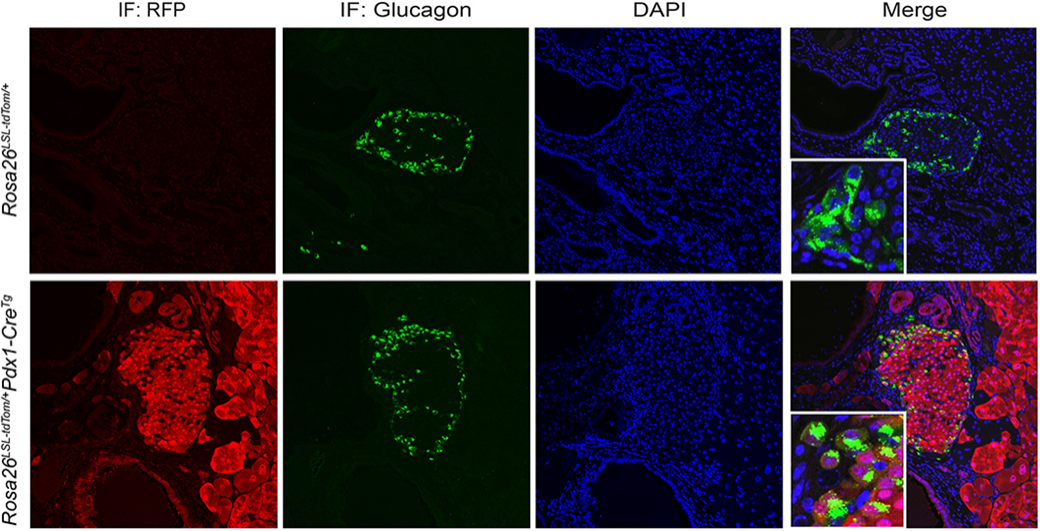
**Immunofluorescence analysis of co-expression of glucagon (α-cell marker) and tdTomato in the pancreas of *Rosa26^LSL-tdTomato/+^Pdx1-Cre^Tg^* mice.** Powerful genetic tools enable us to manipulate gene expression in a temporal and cell-type-specific manner in mice. Genetically engineered mice serve as a valuable model system for many diseases. However, there are still some limitations in this model animal.

**What has surprised you the most while conducting your research?**

*DAXX* and *ATRX* are frequently mutated genes, occurring in 43% of PanNET cases. The human genetics strongly suggested that the two proteins encoded by these genes act as tumour suppressors for PanNETs. However, Daxx and Atrx are not robust tumour suppressors in murine endocrine pancreas, and it is not easy to build a new disease models driven by the mutations.

**Describe what you think is the most significant challenge impacting your research at this time and how will this be addressed over the next 10 years?**

I would say the most significant challenge is that we still don't have enough knowledge on the protein functions of DAXX and ATRX. This lack of knowledge emphasizes the need for basic research. In my opinion, it is important to pay more attention to, and provide more funding for, basic research, where the immediate impact might not be obvious.

**What changes do you think could improve the professional lives of early-career scientists?**

I feel fortunate to be trained in a very supportive and collaborative environment. I would like to give a shout-out to my mentor, my lab, my department and my programme. People there provide enormous support to me, and I can ask anyone for guidance and help at any moment. In my opinion, this kind of friendly and inspiring environment is crucial for the scientists in their early career, when they need lots of support and guidance. Moreover, this environment will help us, young scientists to build confidence, enjoy the work more and have faith in the scientific community when we are facing the challenges along the way.

**What's next for you?**

As I have nearly completed my graduate training, I am interested in pursuing postdoctoral training to prepare myself for an independent research career. I look forward to learning new things and making a contribution to the scientific field.
